# Awareness of food allergies: a survey of pediatricians in Kuwait

**DOI:** 10.1186/s12887-016-0773-9

**Published:** 2017-01-11

**Authors:** Waleed Al-Herz, Khalid Husain, Ahmed Al-Khabaz, Mohamed A. A. Moussa, Fawaz Al-Refaee

**Affiliations:** 1 Department of Pediatrics, Faculty of Medicine, Kuwait University, P.O. Box: 24923, Safat, 13110 Kuwait city, Kuwait; 2 Allergy and Clinical Immunology Unit, Department of Pediatrics, Al-Sabah Hospital, Kuwait City, Kuwait; 3 Gastroenterology Unit, Department of Pediatrics, Al-Ameri Hospital, Kuwait City, Kuwait; 4 Allergy and Clinical Immunology Unit, Department of Pediatrics, Mubarak Hospital, Kuwait City, Kuwait; 5 Department of Community Medicine & Behavioral Sciences, Faculty of Medicine, Kuwait University, Kuwait City, Kuwait; 6 Gastroenterology Unit, Department of Pediatrics, Adan Hospital, Kuwait City, Kuwait

**Keywords:** Diagnosis, Epidemiology, Food allergy, Knowledge, Survey

## Abstract

**Background:**

Early diagnosis of food allergies (FA) is important for a favorable prognosis. This study aimed to determine the level of awareness of FA among pediatricians in Kuwait.

**Method:**

A 43-item self-administered questionnaire was designed and distributed to pediatricians working at 4 government hospitals in Kuwait.

**Results:**

A total of 140 pediatricians completed the questionnaire, with a participation rate of 51.1% (81 males and 59 females). The mean age of participants was 40.81 years, and the mean number of years working in pediatrics was 13.94 years. The mean overall knowledge score was 22.2. The pediatricians’ overall knowledge scores were found to be significantly associated with their age (older pediatricians had higher overall scores) and years of experience as a pediatrician but were independent from hospital site, gender, or rank. A multiple linear regression revealed pediatrician age and gender were the only variables that were significantly associated with the overall knowledge score. Only 16.4% of the participants answered at least 2/3 of the survey questions correctly. The questions that were correctly answered by ≤ 2/3 of the participants constituted 80% of clinical presentation questions, 66.6% of diagnostics questions, 77.7% of treatment questions, and 42.8% of prevention questions. Interestingly, among 68 pediatricians (48.5%) who determined that they felt comfortable evaluating and treating patients with FA, only 12 (17.6%) passed the questionnaire.

**Conclusions:**

This survey demonstrates that there is a noteworthy deficiency of pediatricians’ awareness about FA. The implementation of strategies to improve pediatricians’ awareness is critical to diagnose food allergy patients early and improve their health and outcomes.

## Background

Food allergies (FA) are defined as an adverse reaction to food mediated by an immunologic mechanism involving specific IgE mechanisms (IgE-mediated), cell-mediated mechanisms (non-IgE-mediated), or both IgE- and cell-mediated mechanisms (mixed IgE- and non-IgE-mediated) [1]. FA most often begins in the first 2 years of life and can cause serious or even fatal reactions. In recent years, the prevalence of FA and its severity and complexity are increasing worldwide [2–4]. FA may affect approximately 8% of children younger than 3 years of age, while the prevalence in children with FA associated with eczema is estimated to be as high as 30% [5]. Early diagnosis of FA is crucial for a good prognosis and should lead to proper nutritional management. Parent-perceived FA are common in early childhood, with up to one-third of parents reporting one or more adverse food reactions [6, 7]. Families usually rely on pediatricians for early recognition of clinical manifestations of FA, and work-up of these patients often starts before referring the patients to specialists. A recent study showed that the mean time from the first physician visit to the diagnosis of cow milk allergy was almost 4 months [8]. Recent guidelines have been published by the European Academy of Allergy and Clinical Immunology to provide evidence-based recommendations for the diagnosis and management of FA for healthcare professionals [1]. Several surveys about pediatricians’ and medical professionals’ knowledge and practice about FA have been conducted [9–14]. Collectively, they showed deficiencies in knowledge of clinical presentations, appropriate use of diagnostic tests, and prescriptions for needed treatments. However, there is a lack of data about FA in the Middle East.

The primary aim of this study was to determine the level of food allergy knowledge and experience of pediatricians in Kuwait. We also aimed to identify the knowledge deficiency areas that need to be emphasized in order to facilitate the early and proper diagnosis of children with FA.

## Methods

There are 6 government hospitals in Kuwait providing secondary and tertiary care to pediatric patients. A self-administered questionnaire about FA was designed and distributed to pediatricians working at 4 government hospitals during December 2015. Five clinical immunologists and 10 gastroenterologists were excluded due to their expertise in FA. The questionnaire was divided into 5 sections:

- Demographic characteristics [age, gender, work-related variables (rank, years of experience as a pediatrician)]: 4 questions
- Clinical presentation: 10 questions
- Diagnostics: 12 questions
- Treatment: 9 questions
- Prevention: 7 questions

The questions in the clinical presentation, diagnostics, and treatment sections were based on the standards of care and the current practices of both allergists and gastroenterologists. The questions in the prevention section were based on the reports of the Adverse Reactions to Foods Committee of the American Academy of Allergy, Asthma, and Immunology and the National Institute of Allergy and Infectious Diseases-Sponsored Expert Panel [15, 16].

Finally, participants were asked whether or not they felt comfortable evaluating and treating patients with FA.

A correct answer was assigned one point, whereas a wrong or missing answer was given zero points. The overall knowledge score of each pediatrician regarding FA was computed by totaling the correct answers to the questions included in the clinical presentation, diagnostics, treatment, and prevention sections (38 questions in total). Participants were considered “passing” if their score was at least 67% (i.e., they answered at least 2/3 of the questions correctly).

The Statistical Package for Social Sciences (IBM SPSS Statistics 23, IBM Corporation, Armonk, NY, USA, 2015) was used for data entry and analysis. The cut-off level for statistical significance was *p* ≤ 0.05. The frequency distribution of the overall knowledge score of pediatricians regarding FA was tested for normality. The *t*-test was used to compare the mean overall knowledge score in binary socio-demographic characteristics (such as gender), while the one-way analysis of variance was used to compare the mean overall knowledge score in polychotomous variables such as rank and the different hospital sites. Pearson’s correlation coefficient was used to assess the strength of association between the overall knowledge score and the two quantitative variables, age and duration of experience as a pediatrician. To eliminate confounding effects, the multiple linear regression method was used to identify the significant determinants of the overall knowledge score. The included variables were pediatrician age, gender, rank, hospital site, and years of experience as a pediatrician.

The study was approved by the Research and Ethics Committees of both the Health Sciences Center of Kuwait University and the Ministry of Health in Kuwait. Written informed consent was obtained from the pediatricians for inclusion in the study.

## Results

A total of 274 pediatricians were invited to participate in the study, and 140 (51.1%) responded to the questionnaire (81 males and 59 females) (Table 1). Their age (mean ± SD) was 40.81 years ± 8.88 (range 26-67 years), and the mean number of years working as pediatricians was 13.94 years ± 7.96 (range 1–42 years). The distribution of the participants according to their rank was as follows: registrar: 53.6%, senior registrar: 20.7%, specialist: 5%, senior specialist: 7.9%, and consultant: 12.8%.

**Table 1:** Participation rate, pass rate, and knowledge score of pediatricians according to hospitals

	All hospitals	Hospital 1	Hospital 2	Hospital 3	Hospital 4
Participation rate %	51.1	83.7	56.7	21.8	45.6
Pass rate %	16.4	16.1	21.1	15.8	9.5
Knowledge Score (Mean)					
All sections (38questions)	22.2	22.1	22.8	22.5	21.2
Clinical presentation (10 questions)	6.1	6.2	6.1	5.9	6.1
Diagnostics (12 questions)	5.3	5.3	5.0	5.5	5.5
Treatment (9 questions)	6.2	6.2	6.5	6.4	5.5
Prevention (7 questions)	4.7	4.5	5.2	4.7	4.1

**Fig. 1:**
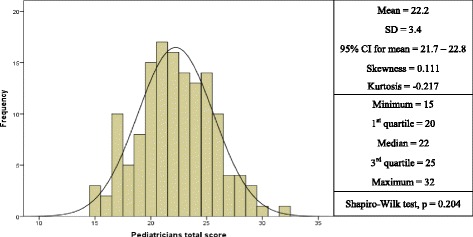
Frequency distribution of overall knowledge scores of pediatricians in Kuwait regarding food allergy

**Fig. 2:**
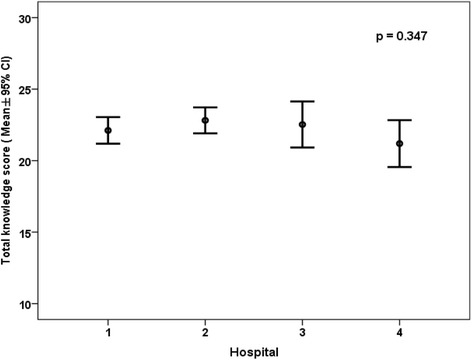
Mean and 95% confidence interval of overall scores of pediatricians in Kuwait regarding food allergy according to hospitals

The overall knowledge score was found to be normally distributed, with a mean of 22.2 (SD = 3.4) and a 95% confidence interval of 21.7 – 22.8 (Fig. 1 and Table 1). There was no significant difference between the mean overall knowledge score and the 4 hospital sites (Fig. 2, *p* = 0.347). The pediatricians’ overall scores were found to be significantly associated with their age (older pediatricians had higher overall scores) (*p* = 0.031) and the number of years of experience as a pediatrician (*p* = 0.003) but were independent from working at any of the hospitals, gender, or rank (*p* = 0.347, 0.106, and 0.38, respectively). Pediatrician age and gender were the only variables that were significantly associated with the overall knowledge score using multiple linear regression analysis (*p* = 0.018 and 0.039, respectively) (Table 2). For every 1 year increase in age, the overall knowledge score would increase by 0.147. Females had a lower knowledge score by −1.373 compared to males.

**Table 2:** Multiple linear regression analysis using total knowledge score as the dependent variable

Variable	Coefficient	*p* value	95% confidence interval
Age (years)	0.147	0.018	0.018 – 0.026
Gender	−1.373	0.039	−2.674 – −0.071
Number of years as a pediatrician	−0.106	0.134	−0.245 – 0.033
Rank	0.298	0.249	−0.212 – 0.809
Hospital	0.226	0.445	−0.358 – 0.811

Tables 3, 4, 5 and 6 show the questions included in different sections of the questionnaire with the percentages of participants who correctly answered them. A significant number of questions were correctly answered by ≤ 2/3 of the participants. These responses constituted 80% of clinical presentation questions, 66.6% of diagnostics questions, 77.7% of treatment questions, and 42.8% of prevention questions.

**Table 3:** Percentage of participants who correctly answered the questions in the clinical presentation section

	Percent
Cow’s milk protein allergy is always accompanied by bloody stools	6.4%
Cow’s milk protein allergy usually presents in the first week of life	16.4%
IgE-mediated food allergic reactions are rapid in onset, typically beginning within minutes to 2 h from the time of ingestion	63.6%
Chronic urticaria is rarely caused by food hypersensitivity	37.1%
Patients with a food allergy almost always have other atopic manifestations	53.6%
Chronic wheezing can be the only manifestation of cow’s milk protein allergy	68.6%
Anaphylaxis caused by food allergy occasionally follows a biphasic course, with a recurrence of symptoms hours after the initial onset	73.6%
Food-related reactions are usually caused by additives/coloring added to the food	57.1%
Behavioral disorders and hyperactivity are frequent manifestations of a food allergy	34.3%
Infantile colic is rarely caused by a food allergy	29.3%

**Table 4:** Percentage of participants who correctly answered the questions in the diagnostics section

	Percent
Skin prick testing can be used to screen patients for an allergy by testing with broad panels of food allergens	72.9%
Skin prick testing is highly sensitive but only moderately specific	66.4%
Intradermal skin testing is recommended for the evaluation of food allergies	58.6%
Atopy patch testing is helpful in diagnostic workup for food allergies	65.7%
Allergen-specific IgE testing is less sensitive than the skin prick test	31.4%
Allergen-specific IgE testing is not useful in patients with severe atopic dermatitis	40.7%
Food challenges should only be performed by allergy specialists familiar with food-allergic reactions	81.4%
Food-specific immunoglobulin G (IgG) and IgG4 tests are additional useful tests for allergies	59.3%
A positive skin test to a particular food indicates that the patient has a true allergy to that food	54.3%
The best way to test for food allergies is by oral challenge	53.6%
Elimination diet should be the first step in the evaluation of a patient presenting with food related anaphylaxis	79.3%
Eosinophilia is an important/common finding in patients with a food allergy	74.3%

**Table 5:** Percentage of participants who correctly answered the questions in the treatment section

	Percent
The only available treatment option for food allergic patients is strict avoidance of the food causing reaction	51.4%
If a patient is diagnosed with food intolerance, he/she may be able to ingest small quantities without having a reaction	53.6%
There is currently no cure for food allergies	55.7%
The most important step in preventing a reaction is parental education regarding avoiding coming in contact with food proteins that can cause an allergic reaction	82.1%
Reading food labels is usually enough to prevent allergic reactions, as they are accurate	17.1%
The prophylactic use of an anti-histamine prevents anaphylaxis in food allergic patients	13.6%
All food allergic patients should be referred to nutritional counseling	69.3%
Cow’s milk allergic patients should be given goat’s or sheep’s milk to avoid nutritional deficiency	17.1%
Patients with a seafood allergy should avoid radio-contrast media used in medical scans due to iodine content	46.4%

**Table 6:** Percentage of participants who correctly answered the questions in the prevention section

	Percent
Dietary restriction during pregnancy and lactation protects against food allergy development in newborn infants	47.9%
Delay of introduction of solid foods after 4 months of age in high risk infants reduces the risk of developing a food allergy	74.3%
Exclusive breast-feeding may help to prevent a food allergy	83.6%
For infants at risk for a food allergy who are not exclusively breast-fed, the use of hydrolyzed infant formulas instead of cow’s milk formula has a preventive effect on atopic disease and cow’s milk protein allergy	90%
Is early introduction of cow milk formula in newborns associated with an increase in the risk of developing a food allergy?	85.7%
It is highly recommended to use probiotics as supplements for neonates as a way to prevent allergies	40%
The use of soy or amino acid formula early in life prevents the development of a food allergy	32.1%

Only 16.4% of the participating pediatricians passed the questionnaire (i.e., correctly answered at least 2/3 of the questions) (Table 1). Interestingly, among the 68 pediatricians (48.5%) who stated they felt comfortable evaluating and treating patients with FA, only 12 (17.6%) passed the questionnaire.

## Discussion

In this study, we determined that the food allergy knowledge and experience of pediatricians in Kuwait were unsatisfactory. We have also identified knowledge gaps that pediatricians should be aware of while caring for patients suspected to have FA.

There were noteworthy deficiencies in pediatricians’ awareness about the clinical presentations of FA, despite this being the cornerstone for early diagnosis. This results in a significant delay in diagnosis and can lead to increased morbidity. A substantial proportion of participants thought that behavioral disorders, hyperactivity, and chronic urticaria were clinical manifestations of FA. This results in unnecessary dietary restrictions and unwarranted referrals to allergists. In addition, more than 1/3 of participants did not know that IgE-mediated food allergic reactions are rapid in onset and can occur within minutes from the time of ingestion, which could predispose patients to a serious risk of anaphylaxis and mortality.

Participants also had major deficiencies in understanding the diagnostic tools used in the work-up of cases suspected to have FA. For example, approximately 60% of participants inappropriately believed that food-specific IgG tests are useful in the evaluation of these patients, despite the clear evidence that these tests have no diagnostic value in the work-up of FA [17]. More than 70% of the participants incorrectly believed that the skin prick test can be used to screen patients for FA by using a broad panel of allergens. Both practices result in an unnecessary financial burden while testing patients who are suspected to have FA. Furthermore, the majority of participants (75%) were incorrect in believing that eosinophilia is a common finding in food allergy patients. Other studies have been conducted that confirm gaps in the knowledge of diagnostics tools used for FA. One study documented a group of pediatricians and primary care physicians in the United States and found that fewer than 30% of the participants felt comfortable interpreting laboratory tests to diagnose FA [8]. Another study also performed in the United States by Bahna et al. documented deficiencies in the knowledge of non-allergists in the methods used for the diagnosis of FA [9].

In the present study, approximately half of the participants improperly believed that patients with seafood allergies should avoid radiocontrast media used in medical scans due to iodine content. A similar number of participants were under the false impression that dietary restrictions during pregnancy and lactation protects against food allergy development in newborn infants, although the current recommendations are clear in this regard. Furthermore, the majority of participants (68%) incorrectly believed that the use of soy or amino acid formula early in life prevents the development of food allergies. These findings suggest that the awareness of pediatricians regarding preventive measures against the development of FA is limited and not up to date. Similar results were also documented in a study performed in Brazil, which concluded that there are gaps in the knowledge of professionals about the primary prevention of FA [10].

Limitations of our study should be noted. First, the questionnaire used was not validated and was not tested for reliability. Second, the authors realize that a passing score of 67% is arbitrary and is not based on scientific background.

## Conclusions

We conclude that pediatricians’ awareness of FA is unsatisfactorily low in Kuwait. It is true that the survey was conducted at a local level; however, we feel that the results are representative of other countries, given the diverse educational background of pediatricians practicing in Kuwait. Therefore, we recommend an implementation of strategies to improve pediatricians’ awareness so that proper interventions and nutritional management can be undertaken to improve patient health and outcomes. These strategies may include comprehensive undergraduate and postgraduate education, organizing continuing medical education (CME) courses, and publishing educational materials (posters, booklets, articles). Special attention should be given to interactive learning by involving pediatricians in the care of food allergy patients. Conducting similar surveys with internists, nutritional specialists, and nurses may provide better data about the awareness of FA among health care providers in Kuwait.

## Abbreviation

FA: Food allergy
